# Eating Disorder Symptoms in Elite Spanish Athletes: Prevalence and Sport-Specific Weight Pressures

**DOI:** 10.3389/fpsyg.2020.559832

**Published:** 2021-01-26

**Authors:** Clara Teixidor-Batlle, Carles Ventura, Ana Andrés

**Affiliations:** ^1^Institut Nacional d’Educació Física de Catalunya (INEFC), Universitat de Barcelona (UB), Barcelona, Spain; ^2^Grup d’Investigació Social i Educativa de l’Activitat Física i l’Esport, Barcelona, Spain; ^3^University of Vic-Central University of Catalonia, Barcelona, Spain; ^4^Faculty of Psychology, Education and Sport Sciences, Blanquerna, Ramon Llull University, Barcelona, Spain

**Keywords:** weight pressures in sport, eating disorder symptoms, prevalence, elite athletes, sport

## Abstract

We determined the prevalence of eating disorder (ED) symptoms among elite Spanish athletes from a broad range of sports and levels of competition and examined the associations between the presence of symptoms and perceived sport-specific weight pressures. We surveyed 646 elite athletes (16.7 ± 4.4 years; 51.08% females) representing 33 sports from top-division teams and two elite athlete training centers in Catalonia. Based on the results of the Eating Attitudes Test-26 responses, 5.1% of athletes (7.6% of females and 2.5% of males) were at risk of EDs. The highest rates of ED symptoms were observed in male endurance athletes and female esthetic athletes. Competition level was not a risk factor. The only gender differences in the presence of ED symptoms by competing level were observed in athletes competing at the national level. Female athletes with ED symptoms scored higher on the two subscales of the Spanish version of the Weight Pressures in Sport (WPS) tool: coach and sport-specific pressures and pressures from teammates and due to uniform. Male athletes with ED symptoms scored higher on the pressures due to uniform subscale. Finally, symptomatic female but not male athletes competing at international and national levels also perceived greater sport-specific weight pressures.

## Introduction

Research from the past 25 years has shown that athletes are at greater risk of developing eating disorders (EDs), than members of the general population (Sundgot-Borgen and Torstveit, 2004; Martinsen and Sundgot-Borgen, 2013). The reported prevalence of ED symptoms among athletes ranges between 6 and 45% in females and 0 and 19% in males (De Bruin, 2017), with variations attributed to differences in populations and methodology (Chapman and Woodman, 2015). The first study to analyze EDs in Spanish athletes detected a prevalence rate of 13% among elite male and female athletes aged 14–25 years from a variety of sports competing at the national level (Pérez Recio et al., 1992). A later study found that 11.4% of young elite Spanish female athletes were at risk for EDs (Toro et al., 2005). More recently, Dosil Díaz et al. (2012) reported a risk rate of 6.8% for Spanish male and female athletes aged from 12 to 52 years.

The sociocultural theoretical model of disordered eating for athletes introduced by Petrie and Greanleaf (2012) holds that athletes face two types of pressures that increase their risk for EDs or disordered eating behaviors: weight and appearance. The model conceptualizes the potential contribution of weight, size, and appearance pressures from coach, teammates, and the wider sporting environment to the development of disordered eating. Sport-specific pressures such as strict weight requirements, revealing sports attire, and esthetic judgments have been found to uniquely contribute to disordered eating beyond the generic sociocultural pressures experienced by the wider population (Petrie and Greanleaf, 2012; Reel et al., 2013). Athletes receive the message that their bodies must not only be perfectly aligned with the societal ideal but also be highly functional so they can excel athletically.

Type of sport, level of competition, and weight-ins can also influence the risk of disordered eating behavior (Galli et al., 2013; Reel et al., 2013; Thompson and Sherman, 2014; Giel et al., 2016), and sport-specific weight pressures may be experienced differently by males and females (Galli et al., 2013; Reel et al., 2013). Female athletes, for example, may feel pressurized to maintain or achieve a low body weight and an ideal body shape. This has traditionally been a slim physique, although there is an increasing trend toward a fitter, more toned body (Kong and Harris, 2015). Male athletes, by contrast, may believe that a greater body weight and muscle mass is advantageous for performance. The “ideal” male body thus would be lean, muscular body that would be well-defined, strong, athletic, and big but not “too big” (Galli and Reel, 2009).

Certain sports are associated with a greater risk of ED development. Early classification systems included six categories (Sundgot-Borgen, 1994): endurance, weight dependence, esthetic, technical, power, and ball sports. Two other categories were later added: motor sports and anti-gravitation (e.g., cross-country skiing and sky jumping) (Sundgot-Borgen et al., 2013). Sports can be further classified as weight-sensitive. These are sports in which leanness or low weight is considered important (Ackland et al., 2012; Giel et al., 2016). They have been linked to higher frequencies of dieting behaviors (Torstveit et al., 2008).

In competitive environments, athletes’ bodies are constantly on display and are judged not only on performance but also on perceived attractiveness. Sports attire thus can heighten athletes’ awareness of their build and accentuate feelings of body shame (Tylka and Hill, 2004; Galli et al., 2013; Cooper and Winter, 2017). Competition attire may exemplify society and sport-related dictations of weight and body shape standards, since a revealing design is often necessary for peak performance (e.g., tight-fitting outfit for cyclists, swimmers, or aerodynamic sprinters) (Greenleaf et al., 2009). Sometimes, however, the criterion is purely esthetic, while in other cases, uniforms may be used as a lure for spectators (e.g., beach volleyball). Self-objectification can thus influence how athletes feel about their bodies (Voelker et al., 2014; Cooper and Winter, 2017).

Competition level is also a risk factor for EDs, with athletes competing at higher levels showing more signs of body dissatisfaction and ED symptoms than those competition at lower levels or non-competitive athletes (Holm-Denoma et al., 2009; Kong and Harris, 2015). Athletes at higher levels of competition are subject to more intense training schedules and greater pressure to conform to an ideal body shape or specific weight, and they are also more prone to perfectionist tendencies (Picard, 1999). Hence, elite athletes are immersed in a competitive, physically demanding weight-conscious culture that can push them to do they believe is necessary to achieve athletic success, which may involve losing weight or increasing muscle mass (Papathomas et al., 2018). Indeed, some athletes and coaches believe that reductions in body weight or fat automatically lead to better performance (Fogelholm, 1994; Manore, 2015). Physiological and psychological changes in developing athletes are another consideration in relation to competition level. In some sports, such as gymnastics, the time frame of peak performance often coincides with adolescence, when elite athletes are required to cope with both the demands of a highly competitive environment and changes associated with physical and sexual growth and maturation (Tan et al., 2016).

Athletes may also experience weight-related pressure from coaches and teammates to achieve a certain weight or body shape that will enhance performance. Coaches are powerful agents in athletes’ lives and can have a considerable influence on the internalization of body ideas and weight goals. Inappropriate coach behaviors, such as public weigh-ins, negative weight-body comments, and expression of high expectations, can also precipitate disordered eating attitudes (Stirling and Kerr, 2012). Coaches are often perceived as experts in anything to do with their sport, including weight loss and nutrition (Reel and Galli, 2006). Their comments and suggestions can be a major source of weight pressure for athletes, as they typically focus on weight and shape rather than on diet or exercise (Coppola et al., 2014).

Teammates can also influence attitudes to body and appearance (Scott et al., 2019). The need to interact with other athletes can lead to socially conditioned body concerns about weight and size. The fact that teammates can report if “someone had put on weight” (Reel et al., 2010), together with the competitive nature of sport, can frequently result in unhealthy body comparisons with teammates, members of rival teams, or successful athletes in their field. Such comparisons, in turn, can lead to unhealthy weight-changing behaviors aimed at achieving an “ideal” weight and body shape/physique for performance (Galli and Reel, 2009). *Fat talks* with teammates and critical or negative comments about body and weight from peers and even parents can cause body concerns (Petrie and Greanleaf, 2012; Reel et al., 2013, 2010).

Anderson et al. (2012) attributed EDs in sport to a combination of sociocultural pressures and sports-specific expectations and demands (Petrie and Greanleaf, 2012; Fortes et al., 2015; Giel et al., 2016). To our knowledge, the last study to analyze the prevalence of EDs among Spanish athletes was published in 2012 (Dosil Díaz et al., 2012) and no studies to date have explored the associations between ED symptoms and body weight pressures in this setting.

The aim of this study was to determine the prevalence of ED symptoms among elite Spanish male and female athletes from a broad range of sports and levels of competition and to investigate associations between the presence of symptoms and perceived weight pressures in sport according to gender, type of sport, and level of competition.

## Materials and Methods

### Participants

A convenience sample of 787 Spanish athletes was recruited, but just 646 were included in the analyses, as 141 (17.9%) did not complete all the questionnaires. Their mean age ranged from 12 to 56 years old (*M*_males_ 16.9 ± 4.7 years; *M*_females_ 16.5 ± 4 years), and they represented 33 sports. Based on the World Health Organization (2020) body mass index (BMI) classification, 1.6% of the athletes were underweight (1% males, 2.2% females), 83.3% were of normal weight (76.7% males, 89.5% females), 13.7% were pre-obese (20.1% males, 7.7% females), and 1.4% were obese (2.3% males, 0.6% females). Mean BMI ranged from 12.1 to 37.6 kg/m^2^ (*M*_males_ 21.8 ± 2.9 kg/m^2^; *M*_females_ 20.7 ± 2.4 kg/m^2^). Descriptive analysis of sample by type of sport according to competition level and gender are shown in Table 1.

**TABLE 1:** Descriptive analysis (%) of sample by type of sport according to competition level and gender.

		Competition level (%)				Gender (%)	
			
Type of sport (%)		International (28.6%)	National (46.9%)	Regional (16.3%)	Missing (8.2%)	Males (48.9%)	Females (51.1%)
Ball sport	50.5%	19.3%	46%	22.7%	12.0%	50.6%	49.4%
Esthetic	17.3%	46.4%	46.4%	7.1%	0%	27.7%	72.3%
Weight category	3.4%	54.5%	22.7%	13.6%	9.1%	45.5%	54.4%
Endurance	18.7%	33.1%	55.4%	7.4%	4.1%	62%	38%
Technical	7.7%	37.5%	37.5%	18.8%	6.3%	50%	50%
Power	2.6%	0%	64.7%	11.8%	23.5%	64.7%	35.3%

**N* = 646.*

### Measures

#### Demographics

A purpose-designed, self-administered questionnaire was used to collect information on age, current weight and height, sport, and level of competition. Self-reported weight and height were used to calculate BMI.

#### ED Symptoms

Eating disorder symptoms were assessed using the Spanish version of the Eating Attitudes Test (EAT-26; Garner and Garfinkel, 1979; adapted to Spanish by Gandarillas et al. (2003). The EAT-26 measures attitudes to eating and ED symptoms and is used to screen for and help diagnosis EDs. The test includes 26 items rated on a 6-point scale. Scores are recoded on a 3-point scale ranging from 0 (*never*, *rarely*, *sometimes*), to 3 (*always*). There are three subscales: (1) *dieting* (13 items; a pathological refusal to consume high calorie and a food preoccupation with physical appearance); (2) *bulimia and food preoccupation* (6 items; episodes of binge eating followed by purging behaviors for body weight loss/control); and (3) *oral control* (7 items; food control and environmental and social forces that promote food intake). The total possible score thus ranges from 0 to 78. A score of 20 or higher indicates clinical risk of EDs. In this study, the Cronbach’s alpha and Omega coefficient for total score were 0.793 and 0.833, respectively.

#### Weight Pressures in Sport

The Weight Pressures in Sport questionnaire for Male (WPS-M; Galli et al., 2013; adapted to Spanish by Teixidor-Batlle et al., 2020) and females (WPS-F; Reel et al., 2013; adapted to Spanish by Teixidor Batlle et al., 2017) was used to assess sport-specific pressures experienced by athletes regarding weight, body shape and size, and appearance. The Spanish WPS-M and the WPS-F have 9 and 15 items, respectively, that are scores on a six-point Likert scale from 1 (*never*) to 6 (*always*). The items in the WPS-F are grouped into two subscales: *pressure from coaches and teammates* (six items) and *pressures due to uniform* (three items). The female version has two subscales: *coach and sport-specific pressures* (eight items) and *pressures from teammates and due to uniform* (seven items). In the present study, the Cronbach’s alpha for the total WPS score was 0.767, and 0.864, for the WPS-M and WPS-F. The corresponding Omega coefficients were 0.770 and 0.873.

### Procedures

The study was approved by the ethics committee of the Catalan government’s Sports Council (06/2016/CEICEGC). Data was collected between October 2017 and February 2018. Athletes from Catalonia’s two elite athlete training centers and clubs with teams competing in the top division in their field division were approached. The researchers contacted the corresponding technical directors or coaches and met with each team to describe the procedure. The athletes were subsequently invited to participate in the study. Data collection in the form of an online survey took place before or after the training session. All athletes provided their informed consent to participate in this study, and anonymity was guaranteed. Families of participants younger than 18 years old were also informed of the nature and purpose of the study and asked to provide signed consent to allowing their children to participate.

The athletes completed the three questionnaires using the *SurveyMonkey*^®^ online survey software on their smart phone. In exceptional cases, paper was used. In all cases, a member of the research team was present during the administration. The survey was administered at the same time to each group of athletes but filled out individually, with no communication allowed. It took approximately 25 min to complete and participation was not remunerated.

### Data Analysis

The statistical analyses were carried out using *SPSS*^®^ (version 24). The reliability of the questionnaires was tested using Cronbach’s alpha and omega. The results of the questionnaires were described as percentages and means and standard deviations. The Mann–Whitney *U* test (U) was used to identify statistical differences according to gender, sport type, competition level, and EAT-26 and WPS scores. The t-test was used to determine differences among BMI between athletes with and without ED symptoms (EAT-26 score < 20 vs. ≥ 20). Differences between genders were analyzed using Fisher’s exact test for the prevalence of ED symptoms and the chi-square test for risk of EDs. Finally, the Kruskal–Wallis test and *post hoc* Dunn comparisons were used to assess differences in mean EAT-26 scores (total and subscale scores) according to type of sport and competitive level. Statistical significance was set at *p* < 0.05.

To achieve sufficient statistical power, the 33 sports were grouped into six categories used in previous research (Sundgot-Borgen, 1994; Giel et al., 2016): endurance, weight category (labeled weight-dependent in previous studies), esthetic, technical, power, and ball sports. Athletes were divided into these groups and also classified as having ED symptoms (at risk) or not (not at risk). As some athletes did not know their competition level, only those who reported competing at the regional, national, or international level were included in the competition-level analysis and comparisons.

## Results

### Eating Disorder Symptomatology

Based on the EAT-26 cutoff score of 20, 5.1% of athletes (*n* = 33) were identified as being at risk for EDs (symptomatic); 75.8% of them were females. Twenty-five of the 330 female athletes (7.6%) had symptoms versus eight of the 316 male athletes (2.5%). Significant differences in the total EAT-26 score were found between athletes with and without symptoms [χ^2^ (1) = 520.7, *p* < 0.001]. Compared with males, females were significantly more likely to have ED symptoms (*p* < 0.05), and they also had a higher total EAT-26 score and a higher dieting subscale score (4.3 ± 5.3 vs. 2.5 ± 3.7, *p* < 0.001). No significant gender was observed for the other two subscales. Overall, 1.9% of athletes competing at the regional level, 6.9% of those competing at the national level, and 4.9% of those competing at the international level had ED symptoms. No differences in the prevalence of symptoms were found according to level of competition. On comparing the results by gender, we found that 3.3% of males and 6.4% of females competing at international level were at risk for EDs. The respective rates for males and females were 2.7 and 10.8% for those competing nationally and 2 and 1.9% for those competing regionally. The only significant gender differences detected for the prevalence of ED symptoms was in groups of athletes competing at the national level [*G*^2^(1) = 8.28, *p* < 0.005]. No differences were found for ED risk according to age (< 18 vs. ≥ 18 years) among male (2.6 vs. 2.5%) or female athletes (7.6 vs. 7.5%). The mean ages of males and females with and without symptoms were similar. ED symptoms were more likely in female (but not male) athletes with a higher BMI [*t*(327) = −2.98, *p* < 0.05; 22.1 ± 2.6 vs. 20.6 ± 2.3 kg/m^2^] and in female athletes competing in weight category sports [*t*(10) = −2.97, *p* < 0.05; 25 vs. 19.1 ± 1.9 kg/m^2^] and endurance sports [*t*(44) = −2.93, *p* < 0.05; 24.7 ± 3.4 vs. 20.7 ± 2.2 kg/m^2^].

Eating disorder symptoms were detected in 5.3% of the male endurance athletes and 2.4% of ball sports athletes. Males in other sport categories did not have an increased risk of EDs. Male weight category athletes had the highest total EAT-26 score (7 ± 5), but no significant differences in total scores were found between athletes in the six categories [χ^2^(5) 3.77, *p* = 0.600] (Table 2). Significant differences were, however, found in the bulimia subscale scores [χ^2^(5) 15.1, *p* < 0.01]. Males in the weight category also scored highest on this subscale (1.8 ± 3). No differences were found among male athletes for total EAT-26 and subscale scores by competition level.

**TABLE 2:** Mean total EAT-26 and subscale scores according to type of sport and gender.

Female athletes	Ball (*n* = 161)	Esthetic (*n* = 81)	Weight (*n* = 12)	Endurance (*n* = 46)	Technical (*n* = 24)	Power (*n* = 6)	Kruskal–Wallis	*P*
	(M ± SD)	(M ± SD)	(M ± SD)	(M ± SD)	(M ± SD)	(M ± SD)		
EAT26	66	9.39	8.56.4	10.26.1	9.111.2	7.73.3	27.3	1 < 2**;
								1 < 4***;
								3 > 1*;
								2 < 4**;
								4 > 5 *
Dieting	34	6.16.5	35.1	5.44.3	5.68.1	4.82.7	31.7	2 > 1 < 4***;
								1 < 6 > 3*;
								2 > 3 < 4**
Bulimia	0.81.5	12.2	3.82.6	1.62	1.42.4	1.21.2	30.7	1 < 3 > 5***;
								1 < 4**;
								2 < 4**;
								2 < 3 > 4**;
								1 < 3 > 5***
Oral control	2.22.5	2.22.4	1.71.5	3.33.1	2.12.2	1.70.5	6.5	1 < 4*;
								2 < 4**

**Male athletes**	**Ball (*n* = 165)**	**Esthetic (*n* = 31)**	**Weight (*n* = 10)**	**Endurance (*n* = 75)**	**Technical (*n* = 24)**	**Power (*n* = 11)**	**Kruskal–Wallis**	***P***
	**(M ± SD)**	**(M ± SD)**	**(M ± SD)**	**(M ± SD)**	**(M ± SD)**	**(M ± SD)**		

EAT26	5.66.4	4.83.5	75	6.36.7	43.1	4.74	3.7	–
Dieting	2.53.8	1.92.7	2.72.3	3.14.3	22.4	1.72.7	4.9	–
Bulimia	0.91.8	0.71.5	1.83	1.42.1	0.20.5	0.81.1	15.1	1 < 4**;
								2 < 4*;
								3 > 5 < 1*;
								4 > 5***
Oral Control	2.22.4	2.22.3	2.51.6	1.72.1	1.81.7	2.23	4.9	–

*p < 0.05; **p < 0.01; ***p < 0.001.

*Ball, ball sports; Esthetic, esthetic sports; Weight, weight category sports; Endurance, endurance sports; Technical, technical sports; Power, power sports; Dieting, dieting scale; Bulimia, bulimia and food preoccupation scale; Oral control, oral control scale.*

The prevalence of ED symptoms among female athletes was 13.6% for esthetic sports, 12.5% for technical sports, 8.3% for weight category sports, 6.5% endurance sports, and 4.3% ball sports. Females competing in power sports did not have symptoms. Female endurance athletes had the highest EAT-26 score (10.2 ± 6.1). The total and subscale score types of sport are shown in Table 2. Significant differences in the prevalence of ED symptoms among female athletes were found between the different sports [χ^2^(5) 27.32, *p* < 0.001], with higher rates observed in esthetic, endurance, and weight category sports. Females from esthetic sports were at higher risk of EDs than those from endurance or technical sports. Differences were also observed for the dieting [χ^2^(5) 31.77, *p* < 0.001] and bulimia subscales [χ^2^(5) 30.74, *p* < 0.001]. Athletes from the esthetics category scored the highest on the dieting subscale (6.1 ± 6.5), while those from the weight category scored highest on the bulimia subscale (3.8 ± 2.6). No differences were observed for the total EAT-26 scores according to level of competition. The only difference observed for subscale scores among female athletes competing at different levels of competition was for oral control [χ^2^(2) 6.05, *p* < 0.05]. The highest score in this case was observed among females competing nationally (2.6 ± 2.8).

### Relationship Between Eating Disorder Symptomatology and the Sport-Related Weight Pressures

Differences in sport-specific weight pressures according to the presence or absence of ED symptoms are shown in Table 3. In the case of male athletes, no differences were found for total WPS-M score or for the coach/teammate pressures score. Males with symptoms, however, scored significantly higher on the pressures due to uniform subscale than those without symptoms in which the subscale related to sports clothing differed between males who scored below and above the cutoff point of the EAT-26 (2.3 ± 1.1 vs. 3.6 ± 1; *U* = 452.00, *p* < 0.01). Generally speaking, male athletes at risk of EDs had higher total and subscale WPS-M scores than those not at risk. On analyzing the results by type of sport, symptomatic male athletes competing in ball sports perceived greater pressures due to uniform than those without symptoms (3.5 ± 0.5 vs. 2.2 ± 1; *U* = 92.50, *p* < 0.05). Across all sports types, male athletes at risk of EDs had the highest sport-specific weight pressure scores, although no differences were found. Males at risk of EDs had the highest total WPS-M scores across the sports modalities, but the differences were not significant. No differences were observed for the WPS-M subscales.

**TABLE 3:** Differences in weight pressures in sport scores between athletes with and without eating disorder symptoms.

	Male athletes		Female athletes	
		
	Symptoms (*n* = 8)	No symptoms (*n* = 308)	Symptoms (*n* = 25)	No symptoms (*n* = 305)
WPS-M (M ± SD)	3.6 ± 0.9	3 ± 0.9		
Coach and teammates	3.6 ± 0.9	3.3 ± 1.1		
Uniform	3.6 ± 0.9*	2.3 ± 1.1		
WPS-F (M ± SD)			3.3 ± 1*	2.2 ± 0.8
Coach			3 ± 1.3**	2.2 ± 1
Uniform and teammates			3.7 ± 0.9**	2.3 ± 0.8

*p ≤ 0.01; **p < 0.001.

*Coach and teammates, weight pressures from coaches and teammates; Uniform, body and appearance pressures due to uniform; Coach, coach and sport characteristics-related pressures; Uniform and teammates, weight pressures from teammates and due to uniform.*

Female athletes with ED symptoms had a higher total WPS-F score than those without symptoms (3.3 ± 1 vs. 2.2 ± 0.8; *U* = 1425.00, *p* < 0.001), and they also scored higher on both subscales. Females with symptoms competing in esthetic sports scored higher on the coach/sport-specific pressures subscale than those without symptoms (3.7 ± 1 vs. 2.2 ± 0.9; *U* = 111.50, *p* < 0.001). In the case of the teammates/uniform pressure subscale, higher scores were observed among symptomatic females for four categories: endurance sports (3.7 ± 0.2 vs. 2.5 ± 0.8; *U* = 13.00, *p* < 0.05), esthetics sports (4.3 ± 0.9 vs. 2.6 ± 1; *U* = 82.50, *p* < 0.001), technical sports (3.1 ± 0.5 vs. 1.8 ± 0.6; *U* = 3.00, *p* < 0.05), and ball sports (3.3 ± 0.6 vs. 2.1 ± 0.7; *U* = 110.50, *p* < 0.001). Overall, female athletes at risk of EDs scored higher overall and on both subscales of the WPS-F. In terms of differences by level of competition, higher teammate/uniform pressure scores were observed among symptomatic females competing internationally (3.9 ± 1.3 vs. 2.5 ± 0.8; *U* = 99, *p* < 0.01) and nationally (3.7 ± 0.8 vs. 2.2 ± 0.9; *U* = 237.50, *p* < 0.001). Finally, symptomatic females competing at an international level scored higher than their symptomatic peers on type of coach/sport-specific pressure subscale (4.4 ± 0.8 vs. 2.6 ± 1.1; *U* = 54.50, *p* < 0.01).

## Discussion

Our findings show that 5.1% of Spanish male and female athletes from different sports and levels of competition were at risk for EDs according to the EAT-26. This percentage is higher than rates reported for athletes in Norway (Martinsen and Sundgot-Borgen, 2013) and Spain (Pérez Recio et al., 1992; Toro et al., 2005). However, and similar to that observed for elite Spanish athletes using the CHAD (eating habits in athletes) questionnaire (Dosil Díaz et al., 2012), the breakdown by gender showed a prevalence rate of 7.6% for females and 2.5% for males. Female athletes were thus three times more likely to have ED symptoms than their male counterparts with recent reports from international studies of a higher prevalence of EDs among female athletes (Bratland-Sanda and Sundgot-Borgen, 2013; Giel et al., 2016; Reel et al., 2016).

We also observed variations in the prevalence of ED symptoms according to type of sport. Our findings support claims by Reel (2011) that each sport has a unique set of demands, with appearance- and body weight-oriented sports linked to an increased risk of ED development. The highest prevalence of ED symptoms among male athletes was observed in endurance and ball sports. Endurance sports have been identified as a high-risk sport because of the preference of a low body weight (Currie, 2010). A slim physique is often thought to be advantageous in the setting, possibly leading athletes to engage in pathogenic weight loss behaviors (Thompson and Sherman, 1993) and fight against their body’s natural size and weight (Petrie and Greanleaf, 2012). Our observation of a higher prevalence of ED symptoms among male athletes engaged in ball sports such as football, volleyball, and handball suggests a recent shift in risk profiles (Milligan and Pritchard, 2006; Díaz et al., 2018; Godoy-Izquierdo et al., 2019). Elite ball sports athletes often represent esthetic “models” judged more on their physical appearance than on their performance, and this may have led to a shift to an ideal body shape characterized by lower weight and greater muscularity. Our findings for female athletes support previous reports showing a higher prevalence of ED symptoms among athletes competing in esthetic sports such as skating, gymnastics, diving, and synchronized swimming (Krentz and Warschburger, 2013). Risk factors in this setting include body weight and shape requirements, revealing sports attire and intense body scrutiny (Sundgot-Borgen and Torstveit, 2004). In short, because of the focus on leanness and physical appearance in general, esthetic athletes have an increased risk of developing body image disturbances and disordered eating behaviors (Smolak et al., 2000; Sundgot-Borgen and Torstveit, 2004).

Three types of sports were associated with an increased risk of EDs in our series: esthetic sports, weight category sports, and endurance sports. Athletes from leanness-oriented sports may be at a greater risk because of the belief that a lean physique is key to optimal performance (Ackland et al., 2012; Nowicka et al., 2013; Kong and Harris, 2015; Thiemann et al., 2015; Mountjoy et al., 2018). The higher prevalence of ED symptoms in the above categories is thus consistent with previous research showing that athletes who perceive low body weight to be important for performance may feel pressurized to be thin (Papathomas et al., 2018). We were surprised, however, to see higher ED symptom prevalence rates among male athletes competing in ball games. As Kerr and Dacyshyn (2000) stated, for instance, prepubescent gymnasts face pressure to maximize their careers in the years before puberty strikes, a time when they are usually competing internationally. This situation is much less common among other athletes, such as those competing in ball sports. Our findings regarding the influence of competition level on risk of ED development are in agreement with those of Voelker et al. (2014), who found no differences between athletes competing at European and international levels and those competing nationally or regionally. Although elite athletes competing at higher levels would typically be more motivated to maintain leaner physiques to improve their chances of winning or attaining athletic success, they may also have greater professional health support and psychological support for developing coping skills (Toro et al., 2005). It has therefore been suggested that ED prevention measures should be reinforced in competitive youth sports (intermediate and upper-intermediate levels) because, as seen in our study, the peak time for symptom onset coincides with the specialization phase of sports, which is also when athletes experience increasing competition pressure (Bergeron et al., 2015; Somasundaram and Burgess, 2018), possibly leading to conflicts between natural physiological changes precipitated by puberty and perceived body ideal for the sport in question (Voelker et al., 2017).

A second goal of the study was to investigate associations between the prevalence of ED symptoms and sport-specific weight pressures. The higher pressures due to sport attire observed in male athletes with symptoms may be related to the theory proposed by Ridgeway and Tylka (2005) that men have two primary body image concerns: muscularity and body fat. Behaviors such as constant body monitoring comparisons may increase the feelings of dissatisfaction, which could be heightened by certain uniforms that draw unwanted attention to the body and accentuate esthetic imperfections (Galli et al., 2013). Significant differences were detected in the perceived weight pressures from coaches/teammates and due to uniform between female athletes with and without ED symptoms. It is known that coaches can pressurize athletes to lose or gain weight to improve leanness or muscularity (Galli et al., 2017). As reported by Muscat and Long (2008), negative comments from coaches and teammates can trigger body image disturbances or restrictive eating behaviors. In our series, perceived pressures from coaches were significantly associated with the presence of ED symptoms in female athletes competing in esthetic sports, supporting previous findings (e.g., Coker-Cranney and Reel, 2015). Our observation is also in accordance with Diaz et al. (2019), who found that a considerable number of coaches showed weight-related beliefs, attitudes, and practices that could potentially enhance the risk of EDs among the athletes under their care. At the same time, our findings highlight the important role that pressures from teammates and sports attire seemed to have on ED risk among female athletes engaged in endurance, esthetics, technical, and ball sports, all sports where a low body weight is considered important for performance and where athletes face great pressure to have a lean body shape or are required to wear a tight-fitting or revealing clothes.

Level of competition has been identified as a potential risk factor for EDs. Our results show that perceived weight-related pressures from coaches and teammates and due to uniforms increased with level of competition across the different sports, although these pressures were not significantly associated with ED symptoms in all cases. Although elite athletes would be expected to face greater pressure to maintain a low weight to achieve athletic success, they would also be more likely to receive greater professional and psychological support to cope with stress and anxiety (Rockwell et al., 2001; Toro et al., 2005). As mentioned, thus, ED prevention measures should be reinforced among younger athletes, since ED symptoms tend to be more common during specialization phases and among athletes competing at lower levels, which may also be when athletes feel greater competition pressure and have limited access to professional support (DiBartolo and Shaffer, 2002; Toro et al., 2005).

The current study has some limitations. First, the athletes were from elite athlete training centers and top-division teams in Catalonia, which is just one of Spain’s 17 autonomous communities. Second, although the study covered a wide range of sports, it did not include certain esthetics sports such as rhythmic gymnastics, and athletes from weight category and power sports were also underrepresented. It would be interesting to include rhythmic gymnastics in future studies as it is considered an appearance-oriented sport in which a young age (pre-pubertal) and a lean body shape are considered important for success (Salbach et al., 2007). Finally, the self-reporting nature of the EAT-26 may have resulted in underreporting of ED behaviors, even though we tried to minimize this potential bias by ensuring full anonymity. Weight and height were also self-reported. Future studies could adopt a sequential approach, with objective measurement of weight and height followed by clinical interviews with athletes identified as at risk by a screening questionnaire.

## Conclusion

This study is the first to assess the association between ED symptoms and sport-specific weight pressures among elite female and male athletes in Spain. Our main findings show higher rates of symptoms in female athletes overall, in esthetic sports, and in male athletes competing in endurance sports. Significant differences were observed in the prevalence of ED symptoms according to type of sport and level of competition. We also detected greater perceptions of sport-specific weight pressures among athletes with ED symptoms, although it should be noted that susceptibility to ED symptoms is also influenced by training environment (Sundgot-Borgen et al., 2013).

The identification of ED risk factors provides a useful basis for future research in this area and could guide more targeted prevention strategies for vulnerable athletes. Further research, however, is needed to reach firm conclusions, particularly in the case of underrepresented sports. Our findings highlight the need for ED prevention programs within clubs and federations and interventions targeting sport professionals working directly with athletes to heighten their awareness of the importance of promoting healthy training environment and minimizing exposure weight pressures.

## Data Availability Statement

The datasets generated for this study will not be made publicly available. Requests to access the datasets should be directed to the corresponding author.

## Ethics Statement

This study was reviewed and approved by the Ethics Committee for Clinical Research of the Catalan Sports Council (06/2016/CEICEGC). Written informed consent to participate in this study was provided by the participants or participants’ legal guardian.

## Author Contributions

CV and AA designed the study. CT-B collected the data and wrote the manuscript. CV and AA supervised the method and statistical analysis. All authors reviewed and approved the final manuscript.

## Conflict of Interest

The authors declare that the research was conducted in the absence of any commercial or financial relationships that could be construed as a potential conflict of interest.
